# The role of quantitative imaging in chronic obstructive pulmonary disease

**DOI:** 10.1590/0100-3984.2022.55.5e1-en

**Published:** 2022

**Authors:** Danilo Tadao Wada

**Affiliations:** 1 Radiologist at Hospital das Clínicas da Faculdade de Medicina de Ribeirão Preto da Universidade de São Paulo (HCFMRP-USP) and at Central de Diagnóstico Ribeirão Preto (Cedirp), Ribeirão Preto, SP, Brazil. E-mail: dwada@hcrp.usp.br

It is undeniable that, from a technological point of view, humanity is constantly evolving. The computers, tablets, and even the cell phones that we currently use have a much higher processing capacity than did those that were available in the 1990s. The evolution of data processing capacity is also a constant. That capacity practically doubles every couple of years, closely following the expectations set by Moore’s famous law^(1)^. In line with that evolution, the field of diagnostic imaging has also been progressing. We went from Roentgen’s discovery of the ability to form an image through the use of X-rays, in 1895, to a field of medicine that uses advanced principles, such as the mobilization of proton particles and radiopharmaceutical labeling of molecules that are expressed in tumor cells. Diagnostic imaging has also precluded the need for countless surgeries, which have been replaced by the use of catheters, clips, springs, valve prostheses, and other materials, implanted in patients in a noninvasive manner.

Our diagnostic capacity is high. Due to the high correlation with histopathology, we can now diagnose malignant neoplasms^(2)^-such as hepatocellular carcinoma-and interstitial lung diseases^(3)^-including those with the usual interstitial pneumonia pattern-thus eliminating the need for biopsy in many cases. Those are situations endorsed not only by the radiology community but also by clinical, surgical, and pathology societies, as a result of the growth of the concepts of multidisciplinary treatment and precision medicine, which focus on what is best for a given patient.

Quantitative imaging cannot be omitted from the story of the evolution of diagnostic imaging. Thanks to efforts such as the chronic obstructive pulmonary disease (COPD) Genetic Epidemiology study^(4)^, thoracic imaging is one of the main actors in that arena. Images acquired by computed tomography (CT) play a fundamental role in several stages of the evaluation of patients with COPD. In the initial evaluation, for example, CT findings indicative of the disease precede by years the expression of the classic clinical phenotypes (the predominance of airway disease or emphysema), thus improving the prognostic stratification of patients, as well as helping call attention to the various comorbidities predominant in each COPD profile. Following the same line of reasoning, the quantification of emphysema by CT can be used in order to stratify patients by 10-year mortality risk^(5)^. In a meta-analysis, Yang et al.^(6)^ showed that the finding of emphysema alone on CT images is a good parameter to identify patients at higher risk of developing primary lung cancer. Because the current criteria for including patients in lung cancer screening programs lead to a high rate of false positives and necessitate a large number of tests to achieve a population-level benefit, this might be a finding that changes the recruitment practices for such programs.

The quantitative evaluation of images can provide many benefits. In other contexts, such as lung cancer, it is possible to use data extraction tools derived from basic information (radiomic analysis), thus improving the correlation with the histological type^(7)^; in the context of pulmonary hypertension, it is possible to demonstrate, more precisely, the redistribution of the pulmonary vascular network^(8)^, allowing us to correlate that with the loss of pulmonary function and to determine the extent of involvement by interstitial disease in systemic sclerosis^(9)^.

In the previous issue of Radiologia Brasileira, there was an article in which the authors performed a quantitative evaluation of bronchial alterations and the presence of emphysema in a specific subpopulation of COPD patients with elevated eosinophil counts in peripheral blood^(10)^. Eosinophilic COPD is a current topic in radiology, and discussion of the topic has led to changes in the treatment of this subpopulation. The article cited above was a retrospective study comparing CT findings between patients with eosinophilic COPD and a control group of COPD patients without elevated eosinophil counts in peripheral blood. The authors found no statistically significant difference between the two groups in terms of airway morphology or the severity of emphysema. That result demonstrates that we need additional studies (in the fields of pulmonology, radiology, and pathology) to help us better understand the particular disease presented by the subgroup of COPD patients with an eosinophilic profile.
